# Microbiota Changes in the Musk Gland of Male Forest Musk Deer During Musk Maturation

**DOI:** 10.3389/fmicb.2018.03048

**Published:** 2018-12-11

**Authors:** Yimeng Li, Tianxiang Zhang, Lei Qi, Shuang Yang, Shanghua Xu, Muha Cha, Meishan Zhang, Zhixin Huang, Juan Yu, Defu Hu, Shuqiang Liu

**Affiliations:** ^1^College of Nature Conservation, Beijing Forestry University, Beijing, China; ^2^Research Department, Zhangzhou Pien Tze Huang Pharmaceutical Co., Ltd., Zhangzhou, China

**Keywords:** *Moschus berezovskii*, musk gland, musk, microbiota, 16S-rRNA gene sequencing

## Abstract

The musk gland in an adult male forest musk deer is an organ that synthesizes, stores, and secretes musk, a cream-colored liquid upon initial secretion that gradually transforms into a blackish-brown solid substance upon full maturation. In this study, four healthy adult male forest musk deer were selected and a total of 12 musk samples were collected for analysis. The samples were in three different states depending on the different seasonal collection dates, which were in June, August, and October. High-throughput 16S-rRNA gene sequencing technology was used to detect microbiota changes in the gland. The results indicate that microbial richness gradually declined during the musk maturation process. The microbiota composition between the initial liquid and final solid musk samples was varied significantly (*P* < 0.05). The dominant bacterial phyla were similar at all three stages included Firmicutes, Proteobacteria, Actinobacteria, and Bacteroidetes. However, the abundances were differences in terms of the dominant bacterial genera. PICRUSt analysis showed the highest represented category was “Amino acid transport and metabolism” (24.8%), followed by “Transcription” (22.04%), and “Carbohydrate transport and metabolism” (20.74%). Our findings indicate that the microbiota in the musk gland plays an important role in the maturation process of musk.

## Introduction

The musk gland in an adult male forest musk deer is located between its navel and genitals. It is an organ that synthesizes, stores, and secretes musk, a viscous cream-colored liquid at initial secretion that eventually becomes a blackish-brown solid substance upon full maturation. Musk is a valuable raw material and ingredient in traditional Chinese medicine that is believed to have anti-inflammatory and anti-tumor properties (Cao and Zhou, 2007; Zhang et al., 2009), as well as significant effects on the central nervous system (Wahab et al., 2018) and the cardiovascular system (Fan et al., 2017). It is also used as a fragrance additive (He et al., 2014). Overall, it has high medicinal and economic value. Its main chemical components include macrocyclic ketone compounds, pyridine compounds, steroidal compounds, peptide-protein compounds, fatty acids and ester compounds, inorganic elements, and a complex microbial community (Sun et al., 2005; Li et al., 2016).

Anatomically, the musk gland has an external opening, which enables development of a complex microbiota (Li et al., 2016). Physiologically, gland temperature rises significantly during the secretion season, with an internal temperature reaching approximately 40°C. That being the optimal temperature for enzymatic reactions, it was deduced that the gland is an enzymatic site (Yin and Dai, 1991). Given that the aforementioned conditions are conducive to microbial colonization and mass proliferation, musk may be formed by the combined effects of the gland’s secretion and microbes. The fermentation hypothesis for mammalian chemical communication assumes that fermentative bacteria in the scent glands of mammals generate odorants that can be used by their hosts for communication and that variation in scent gland odors is due to underlying variation in the structure of bacterial communities within scent glands. The intense odor of musk may be produced by microbial fermentation, and musk is the external pheromone of male musk deer (Hawkins, 1950; Feng et al., 1981).

Some scholars believe that microbes participate in the synthesis of chemical signaling substances in mammalian scent glands (Theis et al., 2013). Given the mammalian and microbial co-evolution process, microbes are expected to display a certain level of stability. Hence, it was posited that the processes of microbial colonization, proliferation, and succession in the musk gland may be coordinated with musk secretion and changes in the gland’s internal environment. During this process, microbes also participate in the formation of musk components. However, little is known about microbiota composition and its changes during the process. It is the important basis for understanding the composition of musk as well as its ecological and pharmacological effects. To this end, our study used high-throughput 16S-rRNA sequencing technology to analyze the microbiota richness, diversity and composition in the musk gland during three stages from musk secretion to maturation, namely initial liquid musk (IM), middle semi-solid musk (MM), and final solid matured musk (FM).

## Materials and Methods

### Ethics Approval Statement

This study was carried out in accordance with the recommendations of the Institution of Animal Care and the Ethics Committee of Beijing Forestry University. The protocol was approved by the Ethics Committee of Beijing Forestry University. The collection of musk samples was approved by the Pien Tze Huang Forest Musk Deer Breeding Center.

### Sample Collection

The study was conducted at Pien Tze Huang Forest Musk Deer Breeding Center, located in Fengxian, Shaanxi Province, a region of Qing Ling Mountain at an altitude of 1,200–1600 m (33°–34°N, 106°–107°E). The region is in a warm temperate zone, with an annual average temperature of 11.4°C and annual average rainfall of 613.2–897.1 mm.

Four adult male forest musk deer (3.5–4.5 years old) were selected for the study. These individuals have never been vaccinated, and in the past 6 months had not received anthelmintic or antibiotic treatments. All selected animals appeared healthy and ear tags were used to distinguish each individual. Prior to collecting musk, the musk deer were placed in cages, and the area surrounding the musk gland stoma and experimental tools were sterilized with alcohol. The base of the gland was clamped and a curette was used to rapidly collect musk, after which the forest musk deer were released. Four samples each of the light cream-colored IM, reddish-brown MM, and blackish-brown FM were collected in June, August, and October, respectively. In each month, the time interval of collecting each sample is less than 30 minutes and the collection process of all samples cost less than 3 h. An average of 0.5 g of musk was collected from a deer at each period. All fresh musk samples were placed in a sterile centrifuge tube, labeled with the respective deer’s ear tag number and the collection time, and immediately stored in liquid nitrogen for transporting back to the laboratory. Subsequently, samples were stored at –80°C and DNA was extracted within 1 week.

### DNA Extraction, PCR Amplification, and 16S-rRNA Gene Sequencing

Total bacterial DNA was extracted using the PowerSoil DNA Isolation Kit (MO BIO Laboratories, United States) according to the manufacturer’s protocol. The quality and concentration of the extracted DNA were measured using a Nanodrop spectrophotometer (ND-1000, NanoDrop Technologies, United States). The V3–V4 region of the bacterial 16S-rRNA gene was amplified by PCR (95°C for 5 min, followed by 15 cycles of 95°C for 1 min, 50°C for 1 min, 72°C for 1 min, and 72°C for 7 min) using the primers 338F (5′-ACTCCTACGGGAGGCAGCA-3′) and 806R (5′-GGACTACHVGGGTWTCTAAT-3′) (Dennis et al., 2013). Indexed adapters were added to the ends of the primers.

PCR products were mixed with the same volume of 2 × loading buffer, and electrophoresis was performed on a 1.8% agarose gel for detection. Samples with a bright main strip of approximately 450 bp were chosen and mixed in equidensity ratios. Then, a mixture of PCR products was purified using a GeneJET Gel Extraction Kit (Thermo Scientific). Sequencing libraries were validated using an Agilent 2100 Bioanalyzer (Agilent Technologies, Palo Alto, CA, United States), and quantified using a Qubit 2.0 Fluorometer. Finally, paired-end sequencing was conducted using an Illumina HiSeq 2500 platform (Illumina Inc., San Diego, CA, United States) at Biomarker Bioinformatics Technology Co., Ltd. (Beijing, China).

### Statistical and Bioinformatics Analyses

The overlapping regions between the paired-end reads were merged using FLASH (V1.2.7), and raw reads were quality filtered under specific filtering conditions to obtain the high-quality clean tags on the basis of the QIIME (V1.8.0) quality control process. Sequences that were less than 200 bp in length or that contained homopolymers longer than 8 bp were discarded. Chimera sequences were detected by comparing tags with the reference database (RDP Gold database) using the UCHIME (V4.2) and then removed. The effective sequences were used in the final analysis.

Sequences were grouped into operational taxonomic units (OTUs) using the clustering program UCLUST (version 1.2.22) (Edgar, 2010) against the SILVA bacterial database (Quast et al., 2013) pre-clustered at 97% sequence identity. Taxonomic classifications (kingdom, phylum, class, order, family, genus, and species) were conducted using the online Ribosomal Database Project (RDP) classifier with a confidence threshold of 80% (Wang et al., 2007). Alpha diversity indexes (ACE, Chao1, Shannon, and Simpson) were calculated by QIIME from rarefied samples for richness and diversity indexes of the microbiota. These values were then compared using ANOVA tests in SPSS Statistics 17.0. The ACE and Chao indexes were used to estimate the number of OTUs in the samples; the Shannon and Simpson indexes are common measures of diversity, which reflect richness and evenness of the samples. The greater the Chao or ACE index, the higher the expected species richness of the microbiota. The smaller the Simpson index and the larger the Shannon index, the larger the microbiota diversity.

Non-metric multi-dimensional scaling (NMDS) based on the Bray-Curtis similarities of OTU composition was applied to rank the bacterial communities, and a one-way analysis of similarity (ANOSIM) was performed to determine the differences in bacterial communities among the three groups using R^1^ (Clarke and Gorley, 2006). Metastats software^2^ was used to compare the difference in bacterial abundance between groups (White et al., 2009). Linear discriminant analysis (LDA) effect size (LEfSe) (Segata et al., 2011) was performed to determine the specific microbiota of the three different states of musk. A size-effect threshold of 3.5 on the logarithmic LDA score was used for identify bacterial taxa. Phylogenetic Investigation of Communities by Reconstruction of Unobserved States (PICRUSt) (Langille et al., 2013) was used to predict the function of musk microbiota based on taxonomy obtained from the Greengenes reference database^3^ (DeSantis et al., 2006). The predicted functions were annotated using the Clusters of Orthologous Groups (COG) database. PICRUSt and LefSe were performed online in the Galaxy workflow framework^4^. The raw sequences obtained in this study have been submitted to the NCBI Sequence Read Archive (accession number SRR6902318).

## Results

### Analysis of rRNA Sequencing Results

The Illumina HiSeq sequencing platform was used to amplify and detect 16S-rRNA gene product sequences in the microbiota of musk from the three periods. A total of 4,970,023 high-quality sequences were acquired from four sets each of the IM, MM, and FM groups. 149,133-1,176,115 valid sequences (Mean length = 423.25 bp) were obtained from each sample. The statistical results of the sequencing data of the various samples are shown in Supplementary Table S1, and the distribution of effective sequence length is shown in Supplementary Figure S1.

**FIGURE 1:**
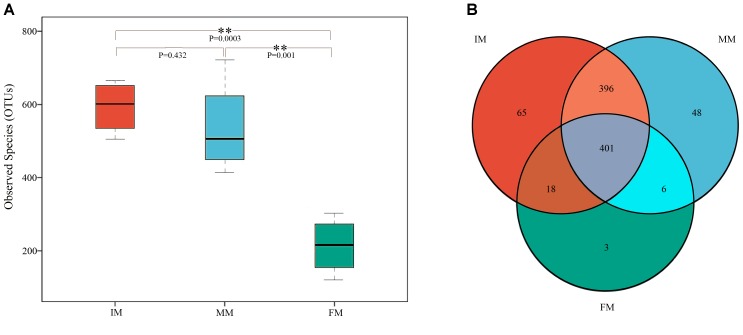
Boxplot of OTUs and Venn diagram. **(A)** Boxplot of OTUs. The *x*-axis shows the different groups and the *y*-axis shows the number of OTUs (observed species). An OTU similarity threshold of 97% was considered. Boxes represent the interquartile range (IQR) between the first and third quartiles (25th and 75th percentiles, respectively), and the horizontal line inside the box defines the median. Whiskers represent the lowest and highest values within 1.5 times the IQR from the first and third quartiles, respectively. ^∗∗^P < 0.01 reflects extremely significant differences. **(B)** Venn diagram. The Venn diagram shows the numbers of OTUs (97% sequence identity) that were shared or not shared by IM, MM, and FM groups (species-level 97% groupings).

The sequences were assigned to 937 OTUs at the similarity threshold of 97% and were then classified using the ribosome database. The detected bacteria could be categorized into 33 phyla, 72 classes, 108 orders, 175 families, and 368 genera. The average number of OTUs obtained per sample was 448 ± 196. The number of OTUs detected in the IM, MM, and FM groups was 593 ± 72.44, 536.5 ± 132.28, and 213.75 ± 77.94, respectively (Figure 1A). The number of OTUs detected in the FM stage was significantly lower than the other two stages (P < 0.01), but the difference in number of OTUs between the IM and MM groups was not significant.

The Venn diagram of the three groups’ OTUs (Figure 1B) shows the number of shared and unique OTUs between groups, providing an intuitive view of the inter-group OTUs matching situation. The number of OTUs shared by all samples within each group was 401, and the number of unique OTUs was 65, 48 and 3 for IM, MM and FM groups, respectively.

### Differences in Microbiota Diversity Among the IM, MM, and FM Groups

Alpha diversity (Ace, Chao 1, Shannon, and Simpson) of the microbiota in the IM, MM, and FM groups was calculated and the results are shown in Table 1. The alpha diversity indexes were calculated based on the OTUs.

**Table 1:** Comparison of alpha diversity indexes of glandular microbiota of the different groups.

Alphadiversity	IM	MM	FM	*P*-value (IM and MM)	*P*-value (IM and FM)	*P*-value (MM and FM)
ACE	632.053 ± 29.837	580.300 ± 98.336	428.716 ± 88.570	*P* = 0.374	*P* = 0.005	*P* = 0.023
Chao 1	638.322 ± 23.840	585.610 ± 98.619	356.403 ± 83.069	*P* = 0.351	*P* = 0.001	*P* = 0.002
Simpson	0.254 ± 0.129	0.240 ± 0.195	0.324 ± 0.110	*P* = 0.892	*P* = 0.528	*P* = 0.446
Shannon	2.261 ± 0.726	2.453 ± 1.775	1.529 ± 0.463	*P* = 0.817	*P* = 0.387	*P* = 0.281


For the IM and MM groups, there was no significant difference for the four indexes. Comparing the MM and FM groups, the results of the ACE and Chao 1 indexes were significantly different (*P* < 0.05), but those for the Simpson and Shannon indexes were not significantly different. For the IM and FM groups, the difference was extremely significant (*P* < 0.01) for the ACE and Chao 1 indexes, but not significant for the Simpson and Shannon indexes.

**FIGURE 2:**
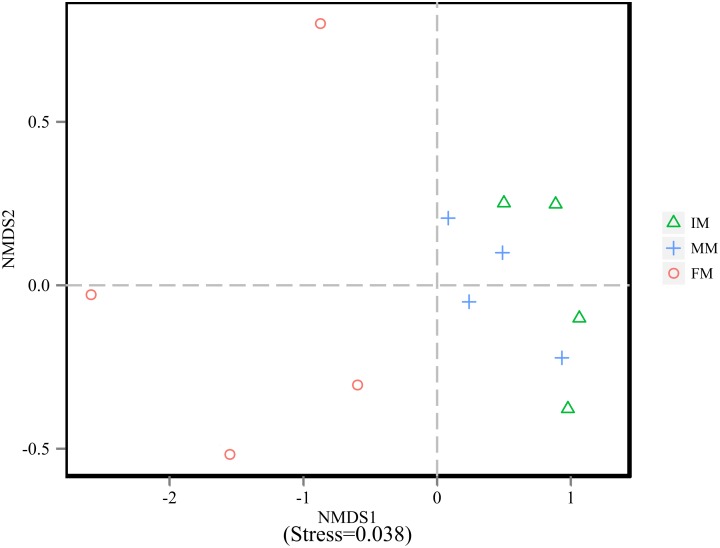
NMDS analysis. Each point represents one sample, and different colors represent different groups. The distance between points represents the level of differences; Stress lower than 0.2 indicates that the NMDS analysis is reliable. The greater distance between two points infers a higher dissimilarity between them.

**FIGURE 3:**
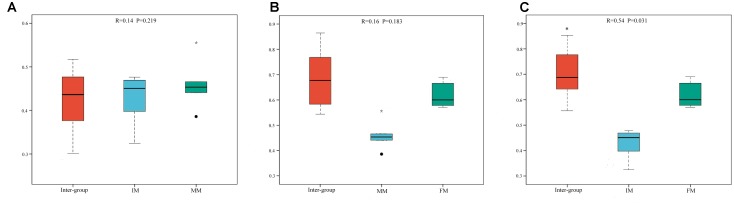
ANOSIM analysis. **(A)** Beta distance of IM and MM. **(B)** Beta distance of MM and FM. **(C)** Beta distance of IM and FM. The *x*-axis represents the grouping and the *y*-axis represents the distance calculated by binary Bray-Curtis. The data in the box is the distance of Inter-group and Intra-group, respectively. R-value: R-value range (–1, 1). An R-value close to 0 represents no significant differences of inter-group and intra-group, and R-value close to 1 shows that inter-group differences are greater than intra-group differences. *P*-value: the *P*-value represents the confidence level of the statistical analysis; ^∗^*P* < 0.05 reflects significant differences between Inter-group and Intra-group. Boxes represent the interquartile range (IQR) between the first and third quartiles (25th and 75th percentiles, respectively), and the horizontal line inside the box defines the median. Whiskers represent the lowest and highest values within 1.5 times the IQR from the first and third quartiles, respectively. “
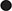
” indicates greater than 1.5 times and less than three times the IQR; “^∗^” indicates greater than three times the IQR.

To analyze the microbiota composition discrepancy between groups, the NMDS plot and ANOSIM analysis was used. The NMDS plot (Figure 2) revealed samples in the IM and MM group tended to cluster together, which showed similarities of bacterial communities between IM and MM group; it also revealed samples in the IM and FM group are separated and showed dissimilarities of bacterial communities between FM and IM group. The ANOSIM analysis is used to test whether there is significant difference in the community composition between groups. Figure 3 revealed significant differences in microbial communities between IM and FM (*R* = 0.54, *P* = 0.031), and negligible differences between IM and MM (*R* = 0.14, *P* = 0.219), MM and FM (*R* = 0.16, *P* = 0.183).

### Microbiota Composition

**FIGURE 4:**
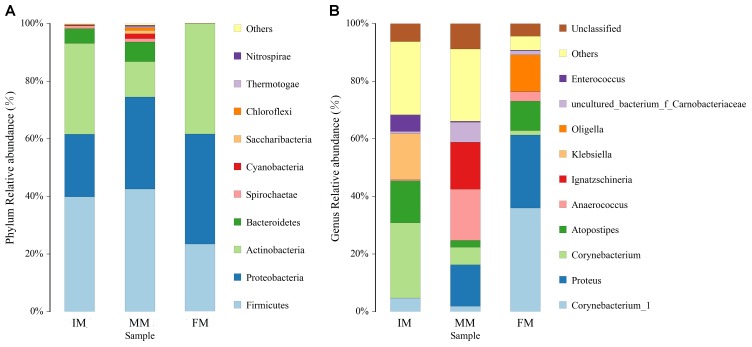
Histogram of relative abundance at phylum and genus levels. The *x*-axis represents groups and the *y*-axis represents relative abundance presented as a percentage. **(A)** Relative abundance of the top 10 phyla. **(B)** Relative abundance of the top 10 genera. Other: Bacterial taxa with ≤ 1% abundance; Unclassified: Sequences which could not be classified.

The top ten bacterial phyla and genera in relative abundance in the IM, MM, and FM groups are shown in Figure 4. Overall, Firmicutes, Proteobacteria and Actinobacteria were the main dominant phyla in IM, MM and FM (Figure 4A). Metastats analysis showed the only phylum with a significantly different relative abundance between the MM and FM groups was Actinobacteria (*P* = 0.008); phyla with significant differences between the IM and FM groups included Bacteroidetes (*P* = 0.048), Spirochaetae (*P* = 0.034), Cyanobacteria (*P* = 0.029), Nitrospirae (*P* = 0.023), and Chloroflexi (*P* = 0.015). At the genera level, the main dominant genera in IM, MM and FM are different (Figure 4B). In IM the main dominant genera is *Corynebacterium*, *Atopostipes* and *Klebsiella*; in MM the main dominant genera is *Proteus*, *Anaerococcus* and *Ignatzschineria*; in FM the main dominant genera is *Corynebacterium 1*, *Proteus*, *Atopostipes* and *Oligella*. Metastats analysis showed between the IM and MM group, *Atopostipes* (*P* = 0.013), and *Corynebacterium* (*P* = 0.039) were significantly different in terms of relative abundance. Between the MM and FM group, *Corynebacterium 1* (*P* = 0.001), *Atopostipes* (*P* = 0.015), and *Anaerococcus* (*P* = 0.037) were significantly different in relative abundance. Finally, significant differences in the relative abundance of *Corynebacterium1* (*P* = 0.003), *Corynebacterium* (*P* = 0.004), and *Proteus* (*P* = 0.008) were observed when IM and FM were compared.

### LEfSe Analysis

**FIGURE 5:**
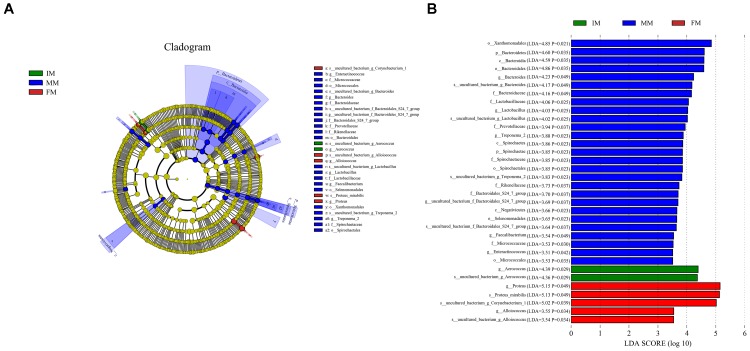
LEfSe analysis. **(A)** The cladogram diagram shows the microbial species with significant differences in the three groups, and the species classification at the level of phylum, class, order, family, and genus shown from the inside to the outside. The red, green, and blue nodes in the phylogenetic tree represent microbial species that play an important role in the three groups, respectively. Yellow nodes represent species with no significant difference. **(B)** Species with a significant difference that have an LDA score greater than the estimated value; the default score is 3.5. The length of the histogram represents the LDA score.

LEfSe analysis was performed to determine differentially abundant bacterial taxa. The cladogram showed differences in 34 taxa among IM, MM, and FM (Figure 5). At the genus level, *Aerococcus* was significantly different between the IM and the other two groups. For the MM group, significantly different genera from the other two groups included *Bacteroides*, *Lactobacillus*, *Treponema2*, *Faecalibacterium*, and *Enteractinococcus*; and significantly different genera between the FM and other two groups were *Proteus* and *Alloiococcus*.

### PICRUSt Analysis

**FIGURE 6:**
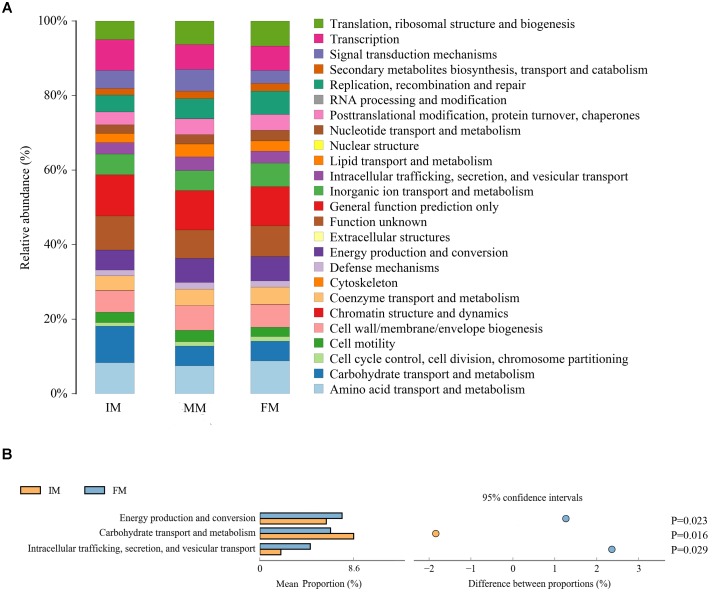
PICRUSt analysis **(A)** COG metagenome functional predictions of OTUs. The *x*-axis represents groups and the *y*-axis represents relative abundance presented as a percentage. **(B)** The abundance ratio of different functions between IM and FM. The middle shows the difference between proportions of functional abundance in the 95% confidence interval, and the value at the rightmost is the *P*-value.

PICRUSt uses the OTU table of assigned taxa to generate the relative abundance of functional categories based on sequenced genomes and annotated using COG database. Predicted abundance of functions (Figure 6A) revealed despite of “General function prediction only” (32.65%) and “Function unknown”(24.85%) categories, the highest represented category at second tier was “Amino acid transport and metabolism” (24.80%), followed by “Transcription” (22.04%) and “Carbohydrate transport and metabolism” (20.74%). Comparing IM with FM, “Energy production and conversion”, “Carbohydrate transport and metabolism” and “Intracellular trafficking, secretion, and vesicular transport” showed significant differences (P < 0.05) (Figure 6B). There was no significant difference category between MM and the other two groups.

## Discussion

Previous studies have shown that the musk gland is a necessary site for the musk maturation process, and that the microbiota might play an important role in maturation and other processes. However, research in this area is currently scarce. Therefore, this study used 16s-rRNA Illumina HiSeq high-throughput sequencing technology to explore microbiota changes during the three stages of the musk maturation process. Our aim was to lay the foundation for further in-depth research of the musk maturation mechanism.

It is generally believed that musk undergoes several processes from secretion to maturation, including the trend of organic and inorganic components becoming stable, reduction in water content, deepening of color, and solidification. These steps result in substantial changes to the microbial living environment within the musk gland. The results show that there was a decreasing trend in the number of microbial species (OTUs) detected over the three stages. The number of OTUs detected in the IM and MM groups was significantly higher than that of the FM group. The ACE and Chao1 indexes of the MM group were significantly higher than that of the FM group, while the same two indexes for the IM group were higher than that for the FM group to an extremely significant level. These data demonstrate that the richness of microbiota gradually decreased during musk maturation. It was proposed that multiple microbes would have the opportunity for bacterial colonization at the initial secretion stage, but many strains were eliminated with changes to the internal glandular environment. Consequently, the level of microbiota diversity was greatly reduced. Other than the musk components, another important factor was possibly microbial competition and exclusion (Lawley and Walker, 2013; Smith et al., 2018). The NMDS plot showed samples in the IM and FM group are separated, together with ANOSIM analysis showed significant differences in microbial communities between IM and FM, and negligible differences between MM and the other two groups. Microbial communities differences may due to the change of the internal glandular environment in different stages.

It was detected from the musk gland’s microbiota that the dominant bacterial phyla were similar for the IM, MM, and FM groups, namely Firmicutes, Proteobacteria, Actinobacteria, and Bacteroidetes. A LEfSe analysis showed that at the genus level, *Aerococcus* was significantly different between the IM and the other two groups. For the MM and the other two groups, genera with significant differences included *Bacteroides*, *Lactobacillus*, *Treponema2*, *Faecalibacterium*, and *Enteractinococcus*; those between the FM and the other groups were *Proteus* and *Alloiococcus*. It is reported that the protein product of *Faecalibacterium* has anti-inflammatory effect (Quévrain et al., 2015), this may be the source of musk anti-inflammatory components; the metabolites of *Bacteroides* contain fatty acids, perhaps this is the source of fatty acids in musk. Moreover, *Bacteroides* and *Lactobacillus* can protect against foreign bacteria invasion, this may be one of the reasons why musk has antibacterial effect (Salyers et al., 2004; Martín et al., 2013). The PICRUSt analysis was used to predict function of the microbial communities. Comparing IM with FM, “Energy production and conversion,” “Carbohydrate transport and metabolism” and “Intracellular trafficking, secretion, and vesicular transport” showed significant differences. We speculate that in FM stage, musk became mature and tends to be stable, with the decrease of bacterial communities, bacteria metabolism became weak. Besides, in this stage, energy production is increase, energy may be stored or used for the synthesis of other components of musk. However, PICRUSt is only a predictor of metagenomic function; thus, further research is required to confirm the accuracy of function information by metagenomic analysis.

Musk components and their changes in the musk gland may result in modifications of microbiota composition. Concurrently, the existence and succession of the bacterial community was also regarded as one of the important factors that cause changes to the musk components (Li et al., 2016). It was hypothesized that there is a complex relationship between musk components and microbial succession and its related metabolic components, with musk being the final product of that complex process. Just as there is a multi-faceted and mutually beneficial symbiotic relationship between an animal’s body and its intestinal microbiota (Collins et al., 2012; Lei et al., 2015; Malys et al., 2015), this study proposed that there may exist a similar mutual and symbiotic relationship between the musk gland and its microbiota. We speculated that the gland may provide essential nutrients for microbial growth, while the microbes probably play an important role in the constitution of musk components. The secondary metabolites of microbes, which may inhibit the growth of other species (particularly harmful or pathogenic ones) (Leclercq et al., 2017), may be the source of antibacterial ingredients in musk. In addition, microbes are able to cause fermentation of carbohydrates to produce short-chain fatty acids (Bäckhed et al., 2004). In turn, fatty acids become the source of the fatty acid components in musk, and the odors of musk may originate from the fermentation process.

Due to the difficulty of sampling, the limitation of our study is the sample size. Firstly, prior to collecting musk, the forest musk deer need to be restrained. However, due to the timid and alert characteristic of forest musk deer, it undoubtedly increases the difficulty of sampling. Therefore, it is difficult to obtain musk samples from individuals with furious reaction. Secondly, the initial experimental design was to collect eight forest musk deer samples from three stages of the secretion period. But because of some individuals having health problems during the sampling period and needed medication, musk samples secreted by them were not taken into account. Excluding these individuals, only four individuals were sampled to obtain a sufficient amount of musk for the experiment (at least 0.5g for DNA extraction). In fact, it is difficult to reach the amount needed at the first time. If too much musk is collect at one time, the forest musk deer will be frightened, leading to a decrease of musk secretion, which will not meet the requirements of continuous sampling in the following stages. Due to the hardships of samples collection and the principles of non-invasive sampling, more specific studies should be conducted further in the future.

In summary, our findings demonstrated that microbiota changes occur during the three stages of musk maturation in forest musk deer. Generally, these findings indicate that the microbiota structure in the musk gland gradually transformed during musk maturation and may played different important roles. Further studies are needed to examine differential microbial roles in the host’s biochemical pathways and physiology, and also required to confirm these hypotheses.

## Author Contributions

YL, DH, and SL conceived and designed the experiments. YL and SX carried out the DNA extraction and data analysis. MC, LQ, and SY participated in the sample collection. YL and TZ wrote the paper. MZ, JY, and ZH assisted with experiments and advice on manuscript content. All authors read and approved the final manuscript.

## Conflict of Interest Statement

The authors declare that the research was conducted in the absence of any commercial or financial relationships that could be construed as a potential conflict of interest.

## Footnotes

1 http://www.r-project.org/

2 http://metastats.cbcb.umd.edu/

3 http://greengenes.secondgenome.com/

4 https://huttenhower.sph.harvard.edu/galaxy/
