# Supplementary material for: Microbiota Changes in the Musk Gland of Male Forest Musk Deer During Musk Maturation
Source: Front Microbiol. 2018 Dec 11;9:3048. doi: 10.3389/fmicb.2018.03048 (PMC6297183; doi:10.3389/fmicb.2018.03048)
Supplement: Supplementary file 1 [file Data_Sheet_1.docx]

Supplementary Material

**Microbiota Changes in the Musk Gland of Male Forest Musk Deer during Musk Maturation**

**Yimeng Li^1^, Tianxiang Zhang^1^, Lei Qi^1^, Shuang Yang^1^, Shanghua Xu^1^, Muha Cha^1^, Meishan Zhang^1^, Zhixin Huang^2^, Juan Yu^2^, Defu Hu*^,1^, Shuqiang Liu^*,1^**

*****Corresponding authors: Defu Hu, hudf@bjfu.edu.cn; Shuqiang Liu, [liushuqiang@bjfu.edu.cn](mailto:liushuqiang@bjfu.edu.cn)

1. Supplementary data

2. Supplementary Figures and Tables

| Sample | PE_reads | Nochimera | AvgLen(bp) | GC(%) | Effective(%) |
| --- | --- | --- | --- | --- | --- |
| IM1 | 525661 | 433412 | 426 | 54.7 | 82.45 |
| IM2 | 425175 | 358881 | 428 | 54.51 | 84.41 |
| IM3 | 403594 | 333212 | 425 | 52.89 | 82.56 |
| IM4 | 359881 | 293378 | 427 | 53.78 | 81.52 |
| MM1 | 632234 | 529413 | 418 | 51.42 | 83.74 |
| MM2 | 1579299 | 1276648 | 430 | 52.34 | 80.84 |
| MM3 | 624790 | 522773 | 419 | 51.09 | 83.67 |
| MM4 | 218974 | 185202 | 418 | 52.88 | 84.58 |
| FM1 | 520335 | 441631 | 429 | 52.65 | 84.87 |
| FM2 | 429482 | 350235 | 417 | 53.45 | 81.55 |
| FM3 | 609773 | 496646 | 422 | 53.72 | 81.45 |
| FM4 | 399421 | 334480 | 420 | 54.2 | 83.74 |

**Supplementary Table S1. Statistical table of post-filtering sequencing data.** Sample: Name of sequencing sample (IM: initial liquid musk; MM: middle semi-solid musk; FM: final solid matured musk); Paired-End (PE) reads: Number of original PE reads; Nochimera: Number of valid sequences after removal of chimeras; AveLen (bp): Average length of valid sequences; GC (%): GC content of valid data; Effective (%): The percentage of valid sequences after chimera removal over the original number of PE reads.


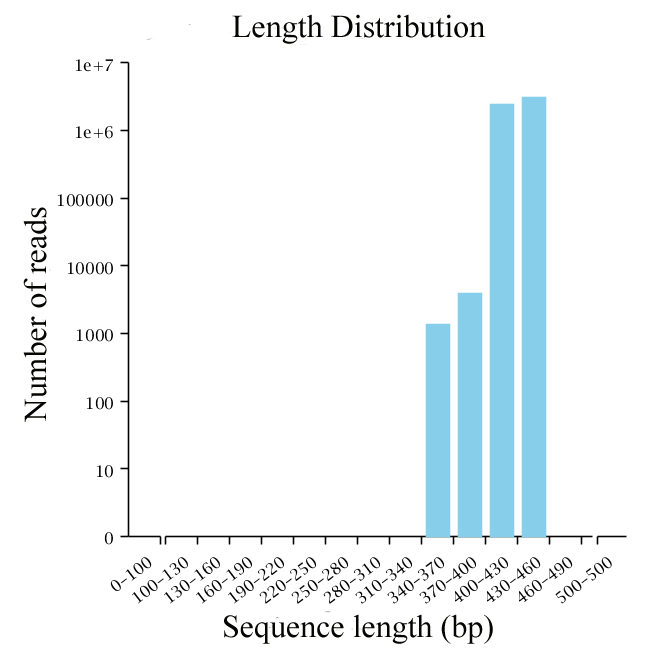


**Supplementary Figure S1. Effective sequence-length distribution.** The X-axis shows the sequence length (bp) and the Y-axis shows the number of reads with each different length (Number of reads).
